# Musicians: Larks, Owls or Hummingbirds?

**DOI:** 10.5334/jcr.173

**Published:** 2019-05-07

**Authors:** Nikita Gjermunds, Inge Brechan, Svein Åge Kjøs Johnsen, Reidulf Gerhard Watten

**Affiliations:** 1 Clinical Psychology, School of Business and Social Sciences, Aarhus University, DK; 2 Department of Psychology, Inland School of Business and Social Sciences, Inland Norway University of Applied Sciences, INN University, NO

**Keywords:** musicians, non-musicians, circadian rhythms, composition, morningness

## Abstract

Previous studies have shown an association between morning and evening types and creative thinking. Musicians are creative individuals and the purpose of the current research was to examine whether musicians are significantly more evening types than non-musicians. The total sample included 835 participants (n women = 353; n men = 482), with a mean age of 28.0 years (*SD* = 10.4). The group of musicians consisted of 600 participants (n women = 168; n men = 432) with a mean age of 29.1 years (*SD* = 11.2). The group of non-musicians consisted of 233 participants (n women = 184; n men = 49) with a mean age of 25.3 years (*SD* = 7.4). Participants were recruited via an online forum, and chronotypes were assessed using the self-report Horne & Ostberg’s Morningness-Eveningness Questionnaire (MEQ). We found that performance musicians had significantly lower MEQ scores compared to non-performance musicians, and musicians who composed had the lowest MEQ scores across the whole sample. This indicates that musicians, particularly composing musicians had a tendency towards eveningness. These findings are discussed in relation to theories on chronobiology, creativity, and cognitive psychology.

## Introduction

Circadian rhythms regulate a number of important functions that are relevant for human beings [1,2]. For instance, they seem to influence physical fitness, sleeping patterns, emotional reactions and complex cognitive functions such as mental organization, planning, and problem solving [3,4]. Circadian rhythms are linked to human biology and genetics, but there is a certain flexibility showing that they can be moderated by culture, socialization and learning [5]. To work as a musician could require performing in the evenings and often late at night. Thus, adaptation to these working schedules over time could induce a lifestyle towards eveningness, where musicians go to bed late at night, but also rise late, clearly favoring evening types (Owls) more than morning types (Larks) and in-between types (Hummingbirds) [1]. In addition, collisions between the musicians’ chronotypes and their external social and working time could occur leading to adverse health effects [6].

There are few studies on the chronotypes of musicians, but research has found interesting associations between creativity and chronotypes. Evening-types (Owls) seem to have elevated scores on several components of creative thinking such as divergent thinking, which may indicate that the Owls’ state of mind deviate from conventional patterns such as applying divergent strategies to visual [7]. Since performing music is a creative activity, and some musicians also create music by being composers, being an Owl would be an advantage. Furthermore, chronotype fluctuations seem to influence timing precisions and technical skills for pianists [8], and a pilot study on professional violinists demonstrated that artistic performance was best between 12 noon and 16:00, and specifically sound instability seemed to be more pronounced in the morning compared to the afternoon [9]. These results suggest that variations in psycho-physiological arousal linked to circadian rhythms is a factor of importance for musicians. Finally, chronotypes and stress reactions should be attended to. Musicians, and particularly professional musicians, take part in concerts and stage performances normally in the evening and late into the night, and concerts and performances with mass media exposure could be experienced as stressful situations for some individuals. Cardiovascular stress reactions and heart rate variability (HRV) are influenced by chronotypes. Compared to morning types, evening types tend to demonstrate elevated heart rate and systolic blood pressure, but lower HRV during stress and baseline [10]. Other research has also suggested an association between eveningness and social stress factors such as severe compulsory internet use and smartphone addiction [11,12], and musicians are frequent users of social media [13]. Together, the findings from these studies indicate musicians’ chronotypes could be of interest also in terms of the relation to stress and health.

In the current study we aimed to address the influences of chronotypes and investigated the self-reported chronotype in a sample of musicians compared to non-musicians. Our primary research question was: Are musicians predominantly Larks, Owls or Hummingbirds?

## Method

### Participants

The total sample consisted of 833 participants (n women = 352; n men = 481) with a mean age of 29.1 years (SD = 10.4). The group of musicians consisted of 600 individuals (n women = 168; n men = 432), the remaining participants being 233 non-musicians (n women = 184; n men = 49). Significantly more men than women reported that they were musicians (Chi-square = 178.69, df = 1, *p* < .001). Of the total sample, 555 participants were also composing music (n women = 158; n men = 397). The majority of those composing music were also musicians (n = 523; n women = 136; n men = 397) and few musicians were not composing n = 77 (n women = 32; n men = 45). Significantly more men than women were composing music (Chi-square = 129.6, df = 1, *p* < .001), also among musicians (Chi-square = 8.1, df = 1 *p* < .01).

### Materials and procedure

We used convenience sampling and all the participants in the study were voluntarily recruited from an established online social media Facebook group called Musicians. Participating musicians and non-musicians were asked two questions:

1. Are you a professional musician or do you regard yourself as a musician? (1 = yes; 2 = no)
2. Have you composed music? (1 = yes, 2 = no)

Since this investigation was centered on musicians and non-musicians as social groups, we did not specify what kind of musical category the participants identified with (e.g. classical, jazz or pop), or the type of musical instruments they were using (e.g. piano, violin or flute).

The questionnaire was available on the Facebook page from February 23th to March 17th, 2015. Prior to the current study, we conducted a pilot-test of the questionnaire in order to estimate the amount of time the participants used to complete the questionnaire. Maximum time was set to 15 minutes, as it was identified if a longer duration the probability of incomplete answers increased. On average participants required 6–7 minutes to complete the questionnaire.

**Assessment of chronotype.** We used the Morningness-Eveningness Questionnaire (MEQ) to assess participants’ chronotype [1]. MEQ is a 19-item questionnaire scored on a Likert type scale from 1 to 4. Questions include: On average, how easy do you find getting up in the morning? (1) not at all easy to 4) very easy; How alert do you feel during the first half-hour after having awakened in the morning? 1) not at all alert to 4) very alert; How is your appetite during the first half-hour after having awakened in the morning? 1) very poor to 4) very good. The MEQ classifies the participants into three basic types: M-type (morning: 59–86), N-type (neutral: 42–58) or E-type (evening: 16–41). The Chronbach’s alpha reliability for the MEQ-scale was .82.

**Statistical analysis.** In addition to descriptive statistics we used Analysis of Variance with Covariates (ANCOVA) in order to test main effects and interactions. The independent variables were group: Musicians, non-musicians, musicians who also composed and non-musicians who composed, and musicians and non-musicians who were not composing. The dependent variable was the MEQ-score. Previous studies have revealed a tendency towards elevated MEQ-scores (increased morningness) for women [14] and older age [15], therefore in our project we controlled for the covariates age and gender in the ANCOVA models. Finally, differences in homogeneity of variances between musicians and non-musicians were tested using Chi-square. The tests showed no significant differences in homogeneity (Chi = 12.9, p = .12).

**Ethics.** The study adheres to the ethical principles of the Helsinki declaration. Participation was voluntary and the questionnaires were responded to anonymously.

## Results

The ANCOVA model controlling for age and gender showed significant differences in chronotypes between musicians and non-musicians *F* (5,827) = 5.3, *p* < .001; partial eta^2^ = .031; observed power = .99. We found a significant main effect of musicians having significantly lower MEQ scores compared to non-musicians *F* (1,827) = 9.4, *p* < .01, and therefore demonstrated a tendency towards eveningness. In addition, we also found a significant interaction with composing music vs. not composing *F* (1,827) = 4.5, *p* < .05, indicating that musicians who composed had lower MEQ scores than those not composing.

We split the sample into two separate groups of musicians and non-musicians and separated these two groups again into two sub-samples: those who composed music and those who did not, since both musicians and non-musicians could make music. Table 1 shows the ANCOVA results for the sub-samples.

**Table 1:** MEQ scores for musicians and not musicians who were composing versus not composing. Multivariate Analysis of Variance with age and gender as co-variates (ANCOVA).

Musicians composing			Musicians not composing			

N	Mean	SD	N	Mean	SD	p

523	43.3	9.2	77	46.8	8.4	<.01
**Non-musicians composing**			**Non-musicians not composing**			

**N**	**Mean**	**SD**	**N**	**Mean**	**SD**	**p**

32	46.6	9.6	201	45.3	9.1	>.05

Musicians who composed music had significantly lower MEQ scores than musicians who did not compose; *F* (1,596) = 8.01, *p* < .01. The mean MEQ scores were 43.3 for composing musicians and 46.8 for non-composing musicians. In the sample of non-musicians there were no significant differences between composing vs. non composing participants: *F* (1,229) = 49, *p* = .48. The mean MEQ values for these groups were 46.5 and 45.2, respectively.

## Discussion

The current study found that musicians had lower MEQ scores compared to non-musicians; i.e. a tendency towards eveningness (Owls). Furthermore, when we examined the sub-samples of composing vs. not composing subjects among both musicians and non-musicians, the composing musicians showed the lowest MEQ scores of all. However, the eveningness category of the MEQ scale comprises of scores in the range of 16–1, and the composing musicians mean value was 43.3. Therefore, composing musicians demonstrated a tendency towards eveningness, they were not clear-cut Owls, but were more late Hummingbirds.

Why should composing musicians show a tendency towards eveningness? One explanation could be linked to creativity and cognitive ability, which seems to be related to eveningness [16]. Creativity can be defined as the ability to produce valuable solutions to problems in new and innovative ways, indicating the importance of divergent thinking compared to convergent thinking [17]. The difference between these two cognitive styles is that through convergent thinking, new information will produce a response to a precisely described situation with an evaluation of a “correct” or “better” solution to the posed problem. In contrast, divergent thinking produces new information more freely by a free-flowing stream of ideas and associations, originality, flexibility, sensitivity for new perspectives and the ability to re-define and re-structure the involved elements. Thus, creativity seems to be predominantly expressed through divergent thinking [7]. Indeed, a study comparing jazz, folk or classical musicians, found that jazz musicians had the highest scores on divergent thinking [18]. Musical improvisations are important in jazz, so it is not surprising that jazz musicians showed elevated scores on divergent thinking domains. The link between creativity and eveningness has also been investigated by a study that examined other central factors of creativity such as mental flexibility, flow, originality and elaboration [7]. The authors found that eveningness individuals had higher scores on all these creative factors. They also found a positive correlation between eveningness and originality, which they defined as the tendency to give unusual answers to questions. Therefore, being awake in late evening and night could stimulate creativity and the ability to find alternative and original solutions to problems [7]. In contrast, there is evidence suggesting that late chronotypes who were tested at subjectively non-optimal times showed increased creative performance than late chronotypes tested at optimal time [19].

It is important to emphasize that the findings of our study do not state that Larks should not become musicians or composers or that Larks are not creative people. Our results show a tendency towards Owl-Hummingbirds for the group of composing musicians, but there is individual variability and Larks could also be excellent musicians and composers. Indeed, Beethoven and Mozart are seminal figures in the history of music, and both of these were early risers [20].

## Strengths and limitations

There seems to be a scarcity of studies on the chronobiology of musicians. We applied a large internet-recruited sample and to the best of our knowledge, is the most comprehensive study in this area so far. However, our results would be strengthened if we obtained more specific data related to the musicians, which could include examining the genre of music individuals performed, such as classical, jazz, folk or pop. It would also be of interest to know more about types of instruments the participants played, such as winds, piano or strings.

## Conclusion

This investigation focused on chronotypes for musicians and non-musicians. The main finding was that musicians, and specifically those who composed music in addition to performing music, had significantly lower MEQ scores than non-musicians. Although these participants showed a tendency towards eveningness, they did not qualify to be classified as evening types, being more late Hummingbirds than Owls.

## Author Informations

Part of this material was earlier used in Nikita Gjermunds’ bachelor thesis (in Norwegian) at the Department of Psychology, Inland Norway University of Applied Sciences, INN University.

## References

[1] Horne, JA and Ostberg, O. A self assessment questionnaire to determine Morningness Eveningness in human circadian rhythms. International Journal of Chronobiology. 1976; 4(2): 97–110. 1027738

[2] Dominoni, DM, Borniger, JC and Nelson, RJ. Light at night, clocks and health: from humans to wild organisms. Biology letters. 2016; 12(2). DOI: 10.1098/rsbl.2016.0015 PMC4780560 26888917

[3] Monk, TH, et al. Circadian rhythms in subjective alertness and core body temperature. Chronobiologia. 1983; 10(1): 49–55. 6851764

[4] Zareian, E, Rabbani, V and Saeedi, F. The Effect of Physical Biorhythm Cycle on Some Physical Fitness Factors of Adolescent Volleyball Players. Annals of Applied Sport Science. 2014; 2(1): 11–20. DOI: 10.18869/acadpub.aassjournal.2.1.11

[5] Finger, FW. Circadian rhythms: Implications for psychology. Psychologist. 1982; 11(1): 1–12.

[6] Keller, LK, et al. Not later, but longer: sleep, chronotype and light exposure in adolescents with remitted depression compared to healthy controls. European child & adolescent psychiatry. 2017; 26(10): 1233–1244. DOI: 10.1007/s00787-017-0977-z 28357513

[7] Giampietro, M and Cavallera, GM. Morning and evening types and creative thinking. Personality and Individual Differences. 2007; 42(3): 453–463. DOI: 10.1016/j.paid.2006.06.027

[8] Van Vugt, FT, et al. The influence of chronotype on making music: circadian fluctuations in pianists’ fine motor skills. Frontiers in human neuroscience. 2013; 7 DOI: 10.3389/fnhum.2013.00347 23847515 PMC3705811

[9] Koch, HJ and Fregin, H. Diurnal variation in performance by orchestral violinists–pilot study. Indian Journal of Physiology and Pharmacology. 2013; 57(3): 242–245.

[10] Roeser, K, et al. Of larks and hearts—morningness/eveningness, heart rate variability and cardiovascular stress response at different times of day. Physiology & behavior. 2012; 106(2): 151–157. DOI: 10.1016/j.physbeh.2012.01.023 22330324

[11] Tonetti, L, Natale, V and Randler, C. Association between circadian preference and academic achievement: a systematic review and meta-analysis. Chronobiology international. 2015; 32(6): 792–801. DOI: 10.3109/07420528.2015.1049271 26125131

[12] Lin, Y-H and Gau, SS-F. Association between morningness–eveningness and the severity of compulsive Internet use: the moderating role of gender and parenting style. Sleep medicine. 2013; 14(12): 1398–1404. DOI: 10.1016/j.sleep.2013.06.015 24157101

[13] Haynes, J and Marshall, L. Beats and tweets: Social media in the careers of independent musicians. New Media & Society. 2018; 20(5): 1973–1993. DOI: 10.1177/1461444817711404

[14] Goel, N, et al. Circadian rhythms, sleep deprivation, and human performance. Progress in molecular biology and translational science. 2013; 119: 155 DOI: 10.1016/B978-0-12-396971-2.00007-5 23899598 PMC3963479

[15] Akerstedt, T and Torsvall, L. Shift work. Shift-dependent well-being and individual differences. Ergonomics. 1981; 24(4): 265–73. DOI: 10.1080/00140138108924850 7195336

[16] Preckel, F, et al. Chronotype, cognitive abilities, and academic achievement: A meta-analytic investigation. Learning and Individual Differences. 2011; 21(5): 483–492. DOI: 10.1016/j.lindif.2011.07.003

[17] Guilford, JP. The nature of human intelligence; 1967.

[18] Benedek, M, et al. Creativity and personality in classical, jazz and folk musicians. Personality and individual differences. 2014; 63: 117–121. DOI: 10.1016/j.paid.2014.01.064 24895472 PMC3989052

[19] Simor, P and Polner, B. Differential influence of asynchrony in early and late chronotypes on convergent thinking. Chronobiology international. 2017; 34(1): 118–128. DOI: 10.1080/07420528.2016.1246454 27791394

[20] Currey, M. Daily rituals: How artists work. Knopf; 2013.

